# Characterization of the Giant Foxtail’s (*Setaria faberi*) *ALS* Gene and Its Enhanced Metabolism-Based Cross-Resistance to Nicosulfuron and Rimsulfuron

**DOI:** 10.3390/genes16050505

**Published:** 2025-04-27

**Authors:** Aristeidis P. Papapanagiotou, Maria V. Alvanou, Ioannis A. Giantsis, Ioannis Vasilakoglou, Ilias G. Eleftherohorinos

**Affiliations:** 1 Department of Agriculture, University of Western Macedonia, 53100 Florina, Greece; 2 Faculty of Agriculture, Forestry and Natural Environment, Aristotle University of Thessaloniki, 54621 Thessaloniki, Greece; malvano@agro.auth.gr; 3 Department of Agriculture-Agrotechnology, University of Thessaly, 41500 Larissa, Greece; vasilakoglou@uth.gr; 4 Department of Field Crops and Ecology, Aristotle University of Thessaloniki, 54621 Thessaloniki, Greece; eleftero@agro.auth.gr

**Keywords:** giant foxtail, *Setaria faberi*, imazamox, nicosulfuron, rimsulfuron, tembotrione, cycloxydim, ALS inhibitors, weed resistance, weed–crop competition

## Abstract

Background: Weed herbicide resistance is a serious problem in crop protection globally. Giant foxtail (*Setaria faberi* R.A.N. Herrm.) populations cannot be controlled by acetolactate synthase (ALS)-inhibiting herbicides in a few corn (*Zea mays* L.) monoculture fields. Methods: Five putative resistant giant foxtail populations, originating from corn monoculture fields in northeastern Greece, were evaluated for possible evolution of ALS-inhibitor resistance (nicosulfuron, rimsulfuron). The resistance ratio, the underlying resistance mechanism, and its impact on competitive ability against corn were studied. Results: The whole-plant rate-response assays showed that these populations were resistant (R) to the sulfonylureas nicosulfuron and rimsulfuron, but susceptible (S) to imidazolinone imazamox, triketone 4-hydroxyphenylpyruvate dioxygenase inhibitor tembotrione, and acetyl-CoA carboxylase inhibitor cycloxydim. The sequencing of the *ALS* gene did not reveal the presence of resistance-associated point mutations, indicating that the resistance was probably not target-site mediated. This was confirmed by the application of piperonyl butoxide two hours before nicosulfuron application, which reversed the resistance in all R giant foxtail populations, supporting the evidence of enhanced metabolism-mediated resistance. The competition study between corn and R or S giant foxtail populations indicated no stable trend reduction in corn traits, suggesting that the resistance mechanism was not associated with the competitive ability of the R populations. The novel *ALS* genotype in *S. faberi*, characterized for the first time and submitted to the GenBank database with accession number PV016837, indicated a closer genetic relationship with the *S. viridis ALS* gene than with *S. italica*. Conclusions: Five giant foxtail populations have evolved metabolism-based resistance to the ALS-inhibiting herbicides nicosulfuron and rimsulfuron.

## 1. Introduction

Giant foxtail (*Setaria faberi* R. A. W. Herrm.) is a member of the *Setaria* genus, which includes green foxtail [*S. viridis* (L.) Beauv.], yellow foxtail [*S. pumila* (Poir.) Roem. & Schult.], bristly foxtail [*S. verticillata* (L.) Beauv.], knotroot foxtail [*S. parviflora* (Poir.) Kerguélen], and foxtail millet (*S. viridis* subsp. *italica*) [1]. Although *Setaria* species are typically temperate, they have been introduced to numerous tropical and subtropical regions, and are regarded as some of the world’s most invasive plants [1,2].

Giant foxtail is a tetraploid (2n = 36) and self-pollinated (autogamous) species that reproduces exclusively through seeds [1]. It has minimal genetic diversity and consistent population structure [3]. This weed is particularly well suited to no-tillage cropping systems [4], as it can germinate on the soil surface, especially when crop residues are present.

*Setaria* species, as fast-growing plants with a C4 photosynthetic pathway, can effectively compete with key agricultural crops, causing significant disruption to farming and land management [5]. Regarding giant foxtail, it is a significant weed in corn (*Zea mays* L.) [6] and soybean [*Glycine max* (L.) Merr.] [1] fields, where it causes substantial yield losses if not controlled, and can also have allelopathic effects on corn [1]. Volenberg and Stoltenberg [7] observed low outcrossing rates in giant foxtail populations, suggesting that seed dispersal is the primary mode of gene flow, rather than pollen movement.

Control of giant foxtail and other *Setaria* species in corn grown in Greece typically involves post-emergence application of ALS-inhibiting herbicides such as nicosulfuron, rimsulfuron, and foramsulfuron, along with the 4-HPPD inhibitor tembotrione. Soil-applied pre-mixtures of S-metolachlor + terbuthylazine or dimethenamid + terbuthylazine are also used for the control of annual grass species in corn. Herbicides that inhibit ALS are particularly prone to resistance evolution, with as few as 10 consecutive applications potentially leading to resistance [8]. Currently, ALS-inhibitor resistance has been reported in 68 grass weed species across diverse global agricultural systems [9].

Target-site resistance (TSR) is the most common resistance mechanism [8], endowed by at least nine amino acid substitutions in highly conserved regions of the *ALS* gene, conferring ALS-inhibitor resistance in weed populations, with 28 different resistance mutations observed in these key residues [10,11]. The TSR could also be attributed to increased expression of the target-site gene (due to regulatory changes increasing transcription and/or increased genomic copy number of the target-site gene), producing more enzyme than can be substantially inhibited by the recommended application rate. Furthermore, a variable number of each gene could occur in polyploid species such as giant foxtail [12,13,14]. Independent or co-existing multiple target-site resistance to ALS- and ACCase-inhibiting herbicides often occurs within the same population [15]. However, although the *ALS* gene has been characterized in other species, this is not the case for giant foxtail, as confirmed by the lack of corresponding sequences in genomic databases like GenBank.

Non-target-site resistance (NTSR) refers to mechanisms that reduce herbicide availability at the target site and may be inherent, induced by abiotic stress, or a combination of both [16]. NTSR poses a significant global threat to agriculture, as it is widespread in grass weed species with resistance to ALS, ACCase, and 5-enolpyruvylshikimate-3-phosphate synthase (EPSPS) inhibitors [17,18]. This mechanism complicates resistance management, as it leads to unpredictable resistance patterns, not only to the herbicides for which resistance evolved, but also to herbicides with different modes of action [19].

It is noteworthy that the coexistence of NTSR and target-site resistance (TSR) mechanisms has already been documented in green foxtail [20] and barnyardgrass [21]. Key genes encoding enzymes responsible for the synthesis of secondary metabolites and the degradation of xenobiotic compounds (e.g., herbicides) can be selected for increased expression and/or field-evolved mutations in weeds due to recurrent herbicide applications. Multiple CYP450 genes are either constitutively expressed or upregulated following ALS-inhibiting herbicide exposure [22]. More specifically, CYP94A1 and CYP71A4 were overexpressed in mesosulfuron-resistant short awn foxtail (*Alopecurus aequalis* Sobol.) populations, while CYP81A12 and CYP81A21 conferred ALS inhibitor resistance in late watergrass (*Echinochloa phyllopogon*). In addition to cytochrome P450s, other enzyme complexes, such as esterases, glutathione S-transferases (GSTs), and uridine 5′-diphospho (UDP)-glucosyl transferases (GTs), have also been implicated in metabolism-based NTSR [22,23].

Resistance to ALS-inhibiting herbicides was first documented in giant foxtail, along with green foxtail and tallow foxtail in the USA and Canada [9]. Resistance in these species has also evolved to herbicides targeting photosystem II, tubulin polymerization, and acetyl-CoA carboxylase, with inheritance patterns varying [24]. Rapid ALS-resistance evolution has been observed in populations repeatedly exposed to ALS inhibitors over four to nine years [25,26]. Point mutations, such as Ser-653-Thr, Ser-653-Asn, Ser-653-Ile, and Gly-654-Asp, have been identified in ALS-resistant green foxtail populations, endowing cross-resistance to ALS-inhibiting herbicides [27,28]. Additionally, resistance to aryloxyphenoxypropionate and cyclohexanedione herbicides (ACCase inhibitors) has been reported in several giant foxtail populations [9,25,28].

Corn farmers in northeastern Greece complained recently about reduced efficacy of the ALS-inhibiting herbicides nicosulfuron and rimsulfuron against giant foxtail populations (personal communication). Based on this information, as well as on the fact that there are no studies investigating the possible fitness cost or the competitive ability of non-target-site-mediated ALS-inhibitor-resistant giant foxtail populations against corn, the objectives of this study were (i) to assess whether five field-selected giant foxtail populations have evolved resistance to nicosulfuron or rimsulfuron and to measure the resistance levels, (ii) to determine the underlying mechanisms conferring resistance, (iii) to evaluate the susceptibility of these populations to herbicides with different modes of action, (iv) to compare the competitive ability of the ALS-resistant populations with that of one susceptible (reference) population against corn, and (v) to analyze the giant foxtail *ALS* gene for the first time.

## 2. Materials and Methods

### 2.1. Seed Source of Giant Foxtail Populations

Seeds of giant foxtail were collected during September 2022 from five corn monoculture fields, located in Kavala, northeastern Macedonia, Greece. This was carried out because farmers in these fields had experienced failure in controlling giant foxtail using sulfonylurea herbicides like nicosulfuron and rimsulfuron. The surviving giant foxtail plants displayed a patchy distribution in areas treated with sulfonylurea herbicides for at least a decade. In order to obtain a representative and adequate sample of each field-selected population, mature seeds were collected by hand from at least 70 to 80 individual plants in each field [29] before corn harvest (September), pooled, and identified as a putative resistant ^®^ population. Additionally, giant foxtail seeds were gathered from an adjacent non-cultivated area that had no history of herbicide use, which was categorized as a potentially susceptible (S) population. After collection, the seeds were brought to the laboratory, air-dried, threshed, placed in paper bags, and stored at room temperature until further use in experiments.

### 2.2. Preliminary Screening and Whole-Plant Rate-Response Assays 

**Figure 1:**
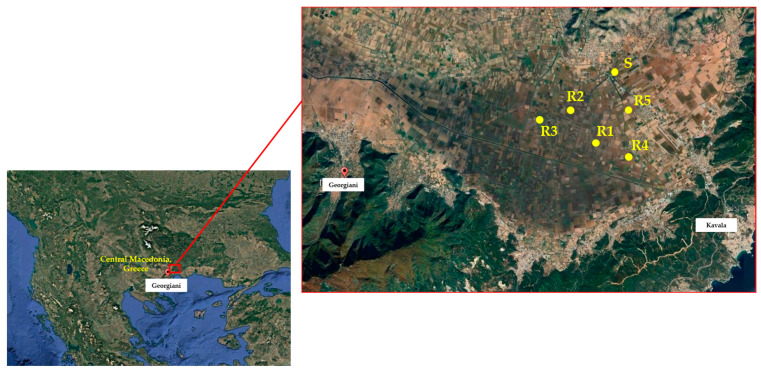
Geographical position of giant foxtail populations evaluated in the present study.

Two separate pot experiments were conducted during the spring and summer of 2023 in a net-protected area at the university farm in Western Macedonia, Greece, to assess the possible evolution of herbicide resistance of giant foxtail populations. In the first experiment, five putative R populations (R1, R2, R3, R4, and R5) originating from corn monoculture fields in Kavala (Figure 1) were tested for their susceptibility to corn-registered herbicides. A putative S population, collected from an untreated area near a corn field, was also included. In particular, these populations were examined for resistance to the sulfonylurea herbicide nicosulfuron, cross-resistance to the imidazolinone herbicide imazamox, and multiple resistance to tembotrione (a triketone 4-HPPD inhibitor) and cycloxydim (a cyclohexanedione ACCase inhibitor). The herbicides were applied at the recommended field label rate (1×) and double the recommended rate (2×) (Table 1) at the two- to three-leaf growth stage (BBCH scale 12–13) of giant foxtail [30]. An untreated control was also included for all populations. As both rates of nicosulfuron provided excellent control of the S population and very low fresh weight reduction in the five putative R populations, these populations were further used in whole-plant rate-response assays.

The five R populations were treated with four nicosulfuron or rimsulfuron rates (1×, 2×, 4×, and 8×) in the whole-plant rate-response assays, while the S population was exposed to sub-doses (1/8×, 1/4×, 1/2×, and 1×) (Table 1). An untreated control was included for each population. Herbicide treatments were applied at the three- to four-leaf growth stage (BBCH scale 13–14) of giant foxtail. Herbicide applications were made using a portable precision field sprayer (AZO-Sprayers, BJ Ede, the Netherlands) with six 8002 flat-fan nozzles, calibrated to deliver 300 L ha^−1^ at 280 kPa pressure. Both experiments were conducted in plastic pots (10 × 10 × 10 cm) filled with a 1:1:1 (*v*/*v*/*v*) clay loam soil, peat, and sand mixture. About 25 giant foxtail seeds were surface-seeded per pot and lightly covered with 3–4 mm of sand. When seedlings reached the two-leaf growth stage, they were thinned to six plants per pot. The plants were irrigated and fertilized as needed throughout the study.

**Figure 2:**
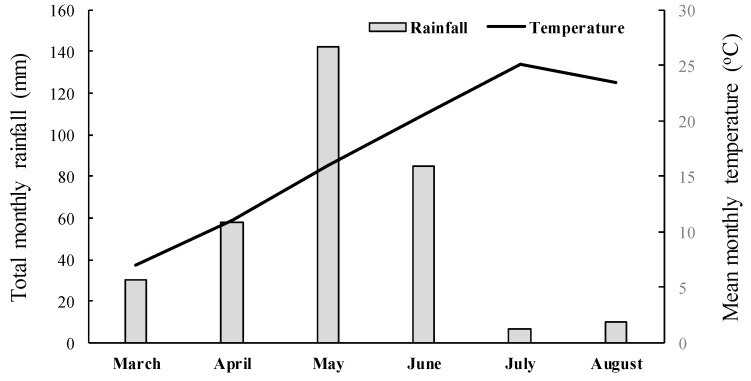
Mean monthly temperature and total monthly rainfall data recorded from March to April 2023 close to the experimental area.

A completely randomized design with three replications per treatment was used in both experiments, and the experiments were repeated in time. The pots were randomized weekly to ensure uniform growth conditions and minimize light variation. Weather data such as total rainfall and average temperature for the experimental area were recorded and are presented in Figure 2. Herbicide efficacy was assessed by measuring the aboveground fresh weight of surviving plants 35 days after treatment (DAT). Fresh weight data were expressed as a percentage of the untreated control and analyzed using ANOVA. Since the whole-plant rate-response assays indicated that the five putative R populations were not controlled by the application of nicosulfuron or rimsulfuron, these populations along with the S one were further used for sequencing of the giant foxtail ALS gene to elucidate the possible existence of a target-site-mediated mechanism of resistance.

### 2.3. Molecular Analysis of the Giant Foxtail ALS Gene

Leaf samples were taken from six surviving plants of each of the five R populations after exposure to the maximum field-label rate of nicosulfuron and from untreated S plants. These samples were stored at −20 °C and subjected to DNA extraction using the NucleoSpin Plant II Mini kit (Macherey-Nagel, Duren, Germany). DNA quality and quantity were assessed with a Q5000 spectrophotometer (Quawell, San Jose, CA, USA). Three primer pairs were used to amplify a 1680 base pair region of the *ALS* gene. More specifically, PCR was performed in total volumes of 20 μL containing 0.6 pmol of one of the forward primers: ALS-1f (5′-GAGATCCACCAGGCGCTAACCC-3′), ALS-2f (5′-TTGGCAACTTCCCCAGTGACGACCC-3′), or ALS-3f (5′-CGGATCAATACACAGTCCTG-3′) and 0.6 pmol of one of the reverse primers: ALS-1r (5′-CAGCTCCTCACCGGATGCAG-3′), ALS-2r (5′-ATGATGGCCTCTCCTTTCGTCAGCTC-3′), or ALS-3r (5′-GATCCGTATTGAGAACCTCC-3′) [31] in various combinations; 10 μL FastGene Taq 2X Ready Mix (Nippon Genetics, Düren, Germany); approximately 40 ng DNA; and water up to the final volume. PCR conditions included an initial denaturation at 95 °C for 3 min, followed by 38 cycles at 94 °C for 30 s, 55 °C for 40 s, and 72 °C for 40 s, with a final extension at 72 °C for 3 min. PCR products were cleaned and sequenced on an ABI Prism 3730 sequencer. Sequences were aligned and analyzed using AliView v. 1.28 [32] and MEGA v. 11 [33] and compared with the green foxtail haplotype (GenBank accession number: KF020514). As the molecular analysis (sequencing) of the giant foxtail *ALS* gene did not reveal the presence of resistance-associated point mutations, the S and the five putative R populations were further used for the metabolism study.

### 2.4. Metabolism Study

To explore the potential role of cytochrome P450 enzymes in ALS-inhibitor resistance in giant foxtail populations, a metabolism study was performed. The pots containing the six giant foxtail populations (one ALS-S and five ALS-R) were prepared in the same way as previously described in the rate-response experiment. Plants at the three- to four-leaf growth stage (BBCH scale 13–14) were treated with piperonyl butoxide [a cytochrome P450 (CYP450) inhibitor] at 2 kg ai ha^−1^. After two hours, nicosulfuron was applied at rates of 1/2×, 1×, and 2× to the S and R populations (R1, R2, R3, R4, and R5). Herbicide applications were conducted with the same equipment used in the rate-response assays. After 35 DAT, fresh weight of the aboveground biomass was recorded and expressed as a percentage of the untreated control. The experiment was performed in a completely randomized design with three replications per treatment and was repeated in time.

### 2.5. Giant Foxtail—Corn Competitiion Study 

**Figure 3:**
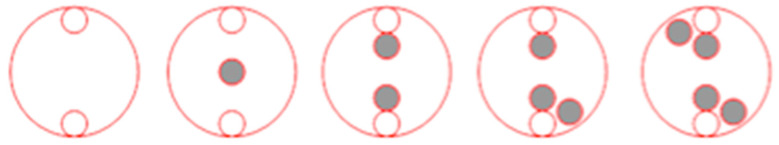
Schematic presentation of the crop: giant foxtail density pattern (2:0, 2:1, 2:2, 2:3, and 2:4) to assess plant responses of corn grown in pure stands and in competition with the R or S giant foxtail populations [corn plants = open circles vs. R or S giant foxtail plants = black circles].

A competition experiment was conducted in the summer of 2023 to assess the competitive ability of three R (R1, R3, and R5) and one S giant foxtail populations against corn (hybrid Hamilton, American Genetics Ltd., Thessaloniki, Greece). Plants were grown in 20 × 25 × 30 cm pots, using a potting mixture of soil, peat, and sand (1:1:1). Giant foxtail and corn seeds were pre-germinated in Petri dishes and then transplanted (at the one-leaf stage) into pots with different plant (corn: giant foxtail) density ratios (2:0, 2:1, 2:2, 2:3, and 2:4). The template used to achieve identical distances between neighboring plants is presented in Figure 3. Pots were placed in a net-protected area for 60 days and received regular irrigation and fertilizer. Weeding was performed to ensure no other weeds competed. The experiment was conducted twice in a completely randomized design with three replicates per treatment. At the end of the experiment, growth parameters such as tiller and spike number for the giant foxtail, as well as height (from soil surface to the leaf tip of the tallest stem) and fresh weight for both corn and giant foxtail, were recorded.

### 2.6. Statistical Analyses

Data from the preliminary screening and rate-response experiments were analyzed using ANOVA with a 2 experiments × 6 populations × 4 herbicides × 2 rates split-plot design for the preliminary screening and a 2 experiments × 5 R populations × 2 herbicides × 4 rates split-plot design for the rate-response experiment. Also, ANOVA was performed for the S population using a 2 experiments × 2 herbicides × 4 rates factorial design for the rate-response experiment. In addition, a factorial ANOVA was used for the metabolism study with a 2 experiments × 6 populations × 7 treatments design. Nonlinear regression analysis was performed on the rate-response data using a four-parameter log-logistic model [34]:

y = c + (d − c)/{1 + exp[b(logx − logGR50)]}(1)

The lower and upper limits were the c and d values, respectively. The b was the relative slope around the herbicide dose, resulting in 50% growth reduction (GR50). The independent variable (x) was the herbicide dose, while the dependent variable (y) was the growth response as a percentage of the herbicide untreated control. The GR50 values were estimated by this equation.

The competition data were analyzed separately for corn and giant foxtail, with a factorial (giant foxtail populations × weed density) ANOVA. The linear regression for growth parameters against weed density was also performed. All analyses were performed using MSTAT-C [35] and R software (version 4.4.1) [36], with significance (Fisher’s protected LSD test) assessed at *p* = 0.05.

## 3. Results

### 3.1. Preliminary Screening and Whole-Plant Rate-Response Results

**Figure 4:**
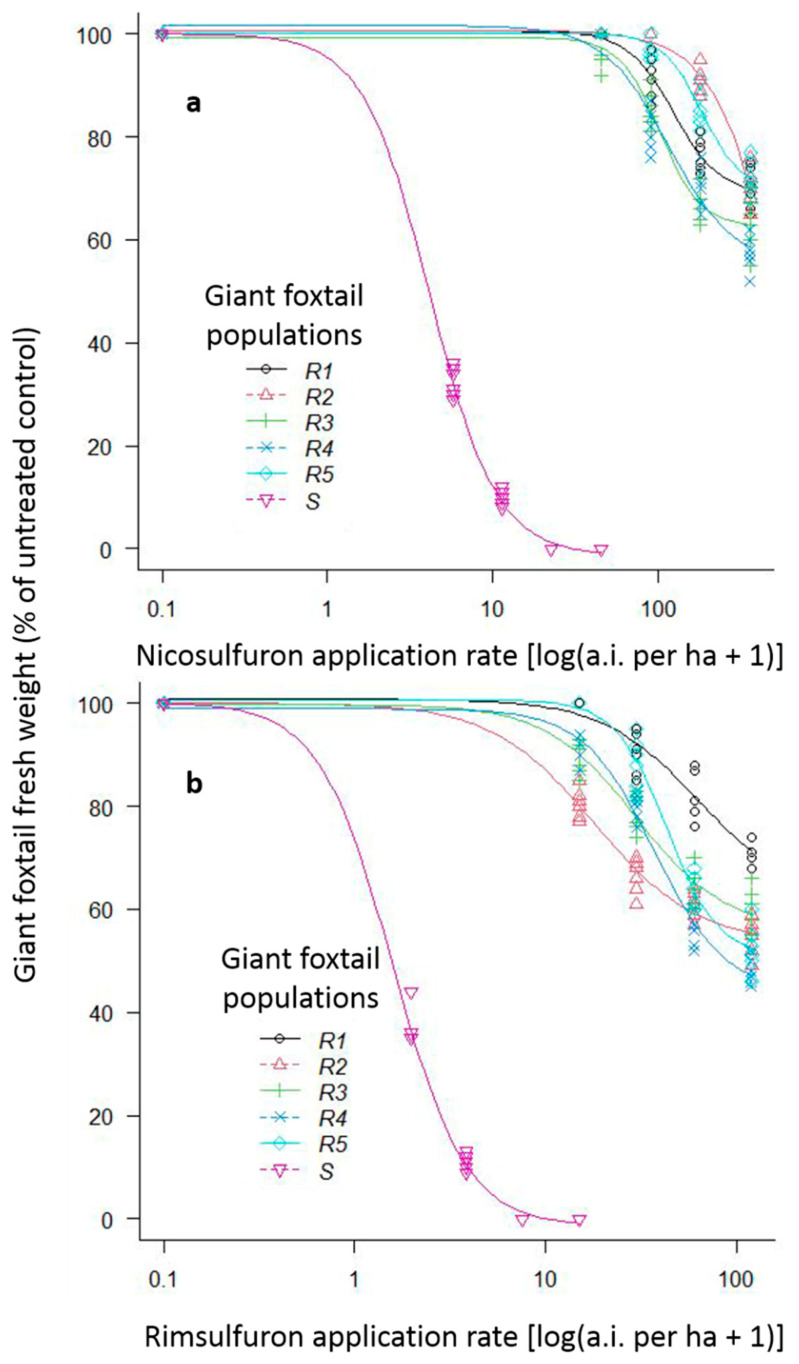
Fresh weight reduction (% of control) of one ALS-susceptible (S) giant foxtail population with the 1/8×, 1/4×, 1/2×, and 1x rates and of five ALS-resistant (R) giant foxtail populations with the 1×, 2×, 4×, and 8× of the label-recommended rate of the ALS-inhibitors nicosulfuron (**a**) and rimsulfuron (**b**).

The ANOVAs conducted for both preliminary screening and whole-plant rate-response assays showed no significant repetition time × treatment interactions. However, both experiments revealed substantial (*p* < 0.001) population × herbicide × rate interactions, and these interaction means are presented in Figure 4.

For the preliminary screening assay, both rates (1× and 2×) of the herbicides imazamox, tembotrione, and cycloxydim provided excellent control (100%) of the six giant foxtail populations. However, both rates of nicosulfuron provided excellent control of the S population but achieved only 0% to 16% fresh weight reduction of the five putative R populations.

The whole-plant rate-response assays showed that the 1/8× rate of nicosulfuron and rimsulfuron reduced fresh weight of the S giant foxtail population by 68% and 63%, respectively, with a 100% reduction achieved with their 1/2× and 1× rates (Figure 4). In contrast, the five putative R giant foxtail populations were poorly controlled by most of the nicosulfuron and rimsulfuron rates used. Specifically, the recommended field-label (1×) and two-fold (2×) rates of nicosulfuron resulted in fresh weight reduction in the five R giant foxtail populations of 0% to 3% and of 0% to 19%, respectively (Figure 4a). The reduction in fresh weight due to nicosulfuron 4x and 8x rates ranged from 9% to 33% and from 28% to 43%, respectively (Figure 4a). Similarly, the application of 1× and 2× rates of rimsulfuron reduced the fresh weight of the R populations by 0% to 20% and by 10% to 34%, respectively (Figure 4b), while the application of the 4× and 8× rates resulted in 17% to 44% and 29% to 52% control of the five R giant foxtail populations, respectively (Figure 4b). In general, the fresh weight of all R giant foxtail populations was reduced more by rimsulfuron compared to by nicosulfuron. This was confirmed by the estimated resistance factors (GR50 of R/GR50 of S) for the R1, R2, R3, R4, and R5 populations, which were higher (29.7, 169.2, 24.7, 27.7, and 43.8) for nicosulfuron than the respective ones for rimsulfuron (39.1, 11.4, 18.8, 23.5, and 27.4) (Table 2, Figure 4).

### 3.2. Characterization and Phylogeny of the Giant Foxtail ALS Gene

**Figure 5:**
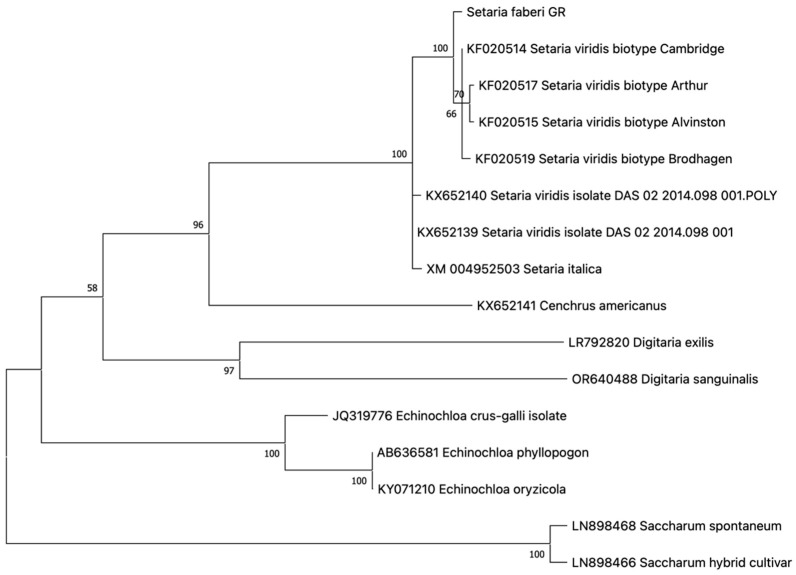
Phylogeny of the *S. faberi ALS* gene in comparison to congeneric and other closely related genotypes. Species, variant, and GenBank accession numbers are shown on each clade. Numbers in branch roots indicate the bootstrap value after 500 iterations.

One haplotype was revealed in all resistant individuals analyzed, indicating 100% homozygosity at the investigated genomic regions. The novel *ALS* genotype in *S. faberi* was characterized for the first time, to our knowledge, and submitted to the GenBank database with accession number PV016837. The maximum likelihood phylogeny of the *S. faberi ALS* gene demonstrated a closer phylogenetic relationship with *S. viridis ALS* than with *S. italica ALS* (Figure 5).

No previously defined resistance-endowing mutation was detected in any of the plants analyzed. However, two nucleotide polymorphisms were identified compared to other *Setaria* genotypes. The first, which was species-specific for *S. faberi*, caused an amino acid substitution from asparagine to glutamine at position 264 of the sequence KF020514. The second, at position 475 of the *ALS* gene, was not species-specific, as it has been observed in other species as well, causing an amino acid substitution from phenylalanine to tyrosine. Both of these polymorphisms were detected in all individuals analyzed, indicating a species specificity for the former, and not supporting any role in resistance.

### 3.3. Metabolism Study Results

**Figure 6:**
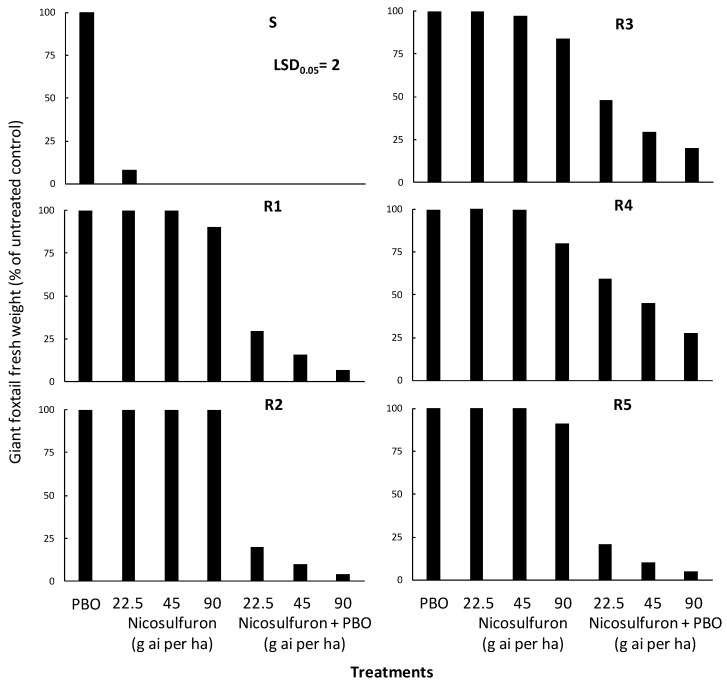
Effect of piperonyl butoxide (PBO) on the efficacy of the ALS-inhibitor nicosulfuron regarding the aboveground fresh weight of one susceptible (S) and five acetolactate synthase (ALS)-inhibitor resistant (PR1, PR2, PR3, PR4, and PR5) giant foxtail populations.

The performed ANOVA indicated significant (*p* < 0.001) population, treatment, and population × treatment interaction, and the interaction means are presented in Figure 4. In particular, piperonyl butoxide (PBO) applied alone did not affect the aboveground fresh weight of all giant foxtail populations (Figure 6). However, PBO applied two hours before nicosulfuron treatments significantly enhanced herbicide efficacy against the five resistant (R) giant foxtail populations. More specifically, the pre-treatment of PBO followed by the application of nicosulfuron at a 1/2× rate caused fresh weight reductions of 70%, 80%, 52%, 41%, and 79% in the R1, R2, R3, R4, and R5 populations, respectively (Figure 6). Similarly, the application of the 1× or 2× nicosulfuron rates after PBO treatment resulted in fresh weight reductions ranging from 84% to 93% in R1, from 90% to 96% in R2, from 70% to 80% in R3, from 55% to 72% in R4, and from 90% to 95% in R5.

### 3.4. Giant Foxtail Competition with Corn

**Figure 7:**
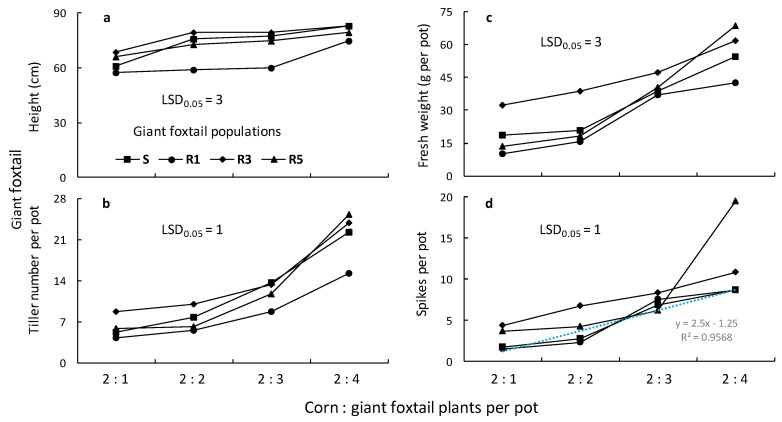
Height (**a**), number of tillers (**b**), fresh weight (**c**), and panicle number (**d**) of one susceptible (S) and three ALS-resistant (R1, R3, and R5) giant foxtail populations as affected by competition with corn.

**Figure 8:**
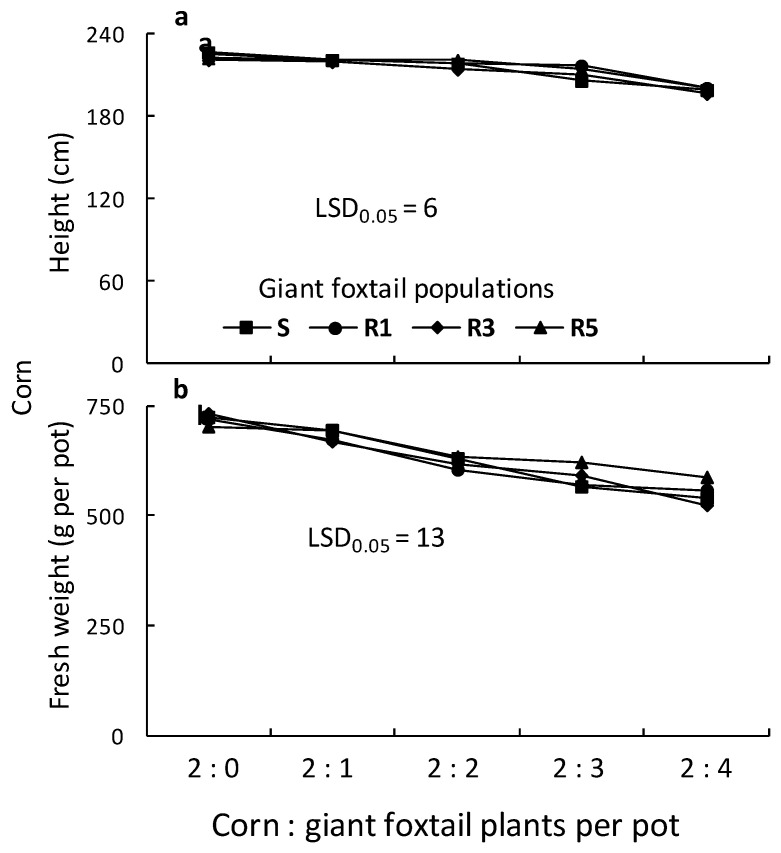
Height (**a**) and fresh weight (**b**) of corn as affected by competition of one susceptible (S) and three ALS-resistant (R1, R3, and R5) giant foxtail populations.

The ANOVAs conducted for the giant foxtail and corn data indicated significant (*p* < 0.001) population, giant foxtail density, and population × giant foxtail density interactions in most cases. Therefore, the means of the population × giant foxtail density interaction are presented in Figure 7 and Figure 8. More specifically, the traits (height, tiller number, aboveground fresh weight, and panicle-spike number) of giant foxtail grown in competition with corn increased with increasing weed density but followed different trends (Figure 7). In particular, the linear equations indicated that the S population had greater slopes for tiller number, fresh weight, and panicle production than the R populations, but a lower slope than the R1 and R3 populations in terms of height (Table 3). These results suggest that the field-selected metabolism-based resistance mechanism conferring a high level of cross-resistance to nicosulfuron and rimsulfuron was not associated with any considerable vegetative output fitness cost under competitive conditions but showed a potential reproductive cost.

The height of corn grown in competition with three R1 giant foxtail plants was reduced by the competition of the S and the other two R populations. However, corn height reduction due to competition from one, two, or four giant foxtail plants per pot was similar for all four populations (Figure 8). The fresh weight of corn plants grown in competition with one or two giant foxtail plants was reduced by 1% to 8% and by 9% to 16%, respectively, across all four giant foxtail populations. However, competition with three or four plants per pot from the R populations caused greater corn fresh weight reductions (19% to 28%) than the S population (12% to 16%).

Overall, corn height and fresh weight decreased with increasing giant foxtail density but followed different trends. This was further confirmed by linear equations estimated from regression analysis between corn height or fresh weight and weed density. The results show that competition from the R1 population caused a greater slope in corn height reduction than competition from the S, R3, and R5 populations, while the fresh weight slope was similar to that of R5 and higher than those of S and R3 (Table 3). Additionally, the slope of corn fresh weight reduction caused by the S population was lower than those of the R populations, suggesting similar competitive ability among the three R populations.

## 4. Discussion

The unsatisfactory control of putative R giant foxtail populations with nicosulfuron applied at the recommended label and two-fold rates in the screening experiment strongly supports the evolution of ALS-inhibitor resistance. The excellent control of all nicosulfuron-resistant populations with herbicides with different modes of action (tembotrione, cycloxydim) indicates that these giant foxtail populations had not yet evolved multiple resistance. Additionally, the excellent efficacy of all herbicide treatments against the putative S population confirms its selection as the reference population.

The evolution of high-level cross-resistance to these herbicides could be attributed to corn monoculture in the studied regions, which resulted in high reliance on single herbicide chemistry that was initially highly effective against target weeds [37]. Giant foxtail populations with ALS resistance to the sulfonylurea herbicides nicosulfuron, rimsulfuron, and primisulfuron-methyl have also been reported in another study [9]. Additionally, other weed species, such as johnsongrass (*Sorghum halepense*), barnyardgrass (*Echinochloa crus-galli*), other foxtails (*Setaria* spp.), and large crabgrass (*Digitaria sanguinalis*), have also evolved resistance to nicosulfuron used for their control in corn [9].

Based on these results, soil-applied herbicides, such as very-long-chain fatty acid (VLCFA)-inhibiting chloracetamides (e.g., S-metolachlor or dimethenamide in premixes with the PS II-inhibitor terbuthylazine), are used as chemical control alternatives to improve early-season weed control and manage ALS resistance in conventional tillage corn fields [38]. However, due to the long emergence window of foxtails and the potential reduced efficacy of soil-applied herbicides under prolonged drought conditions, the triketone herbicide tembotrione (a 4-HPPD inhibitor) is used, as it is less prone to resistance evolution compared to ALS-inhibiting herbicides. Furthermore, considering the high reproductive output and effective seed dispersal mechanisms of giant foxtail, which facilitate rapid field-selected resistance under intense herbicide selection pressure [37], crop rotation and diversification of weed control measures should be implemented to slow or mitigate the evolution of herbicide resistance.

The sequencing of the *ALS* gene did not reveal the presence of resistance-associated point mutations, suggesting that the giant foxtail resistance to nicosulfuron and rimsulfuron was probably not target-site mediated. This finding contrasts with previous studies where target-site-mediated resistance (TSR) to nicosulfuron was detected in populations of the closely related green foxtail. In those cases, resistance resulted from point mutations that caused Asp-376-Glu and Pro-197-Ala amino acid substitutions in the *ALS* gene [31], or TSR co-existing with a non-target-site resistance mechanism mediated by cytochrome P450 monooxygenases [20]. On the other hand, the novel point mutations discovered in the present study cannot be directly associated with resistance, but it should be noted that they have not been identified previously. These results align with Laplante et al. [27], who highlighted the challenge of predicting resistance due to the variability of herbicide selection pressure selecting for point mutations in various weed populations. In the same study, point mutations were detected as leading to various amino acid substitutions such as Ser-653-Thr, Ser-653-Ile, and Gly-654-Asp, which conferred TSR to nicosulfuron as well as chemically dissimilar ALS inhibitors (imazethapyr, flucarbazone, and pyrithiobac) in field-evolved green foxtail populations. It is therefore proposed that while TSR has been widely reported in other *Setaria* species, the resistance mechanism *in S. faberi* may be different and could involve alternative resistance pathways that warrant further investigation.

The fact that the piperonyl butoxide (PBO) reversed the resistance in all R giant foxtail populations supports the evidence of enhanced metabolism-mediated resistance due to Cytochrome P450 monoxygenases. Nevertheless, here we did not perform gene expression analysis to shed more light on this mechanism. Despite this limitation, similar results have been reported by other studies [19,39], which found that the application of piperonyl butoxide (PBO) or organophosphorus insecticides such as phorate and malathion effectively reversed metabolic-based resistance to ALS-inhibiting herbicides in various grass weed species. Field-selected non-target-site resistance (NTSR) in grass weeds, which compromises the efficacy of ALS-inhibiting herbicides, presents an increasing challenge in various crop production systems [17,36]. However, Délye et al. [17] and Wang et al. [19] reported that NTSR is not restricted to ALS herbicides but is widespread across grass weeds, reducing the efficacy of multiple herbicide modes of action, such as the EPSPS inhibitor glyphosate and ACCase inhibitors [16]. The NTSR mechanisms include not only herbicide detoxification but also reduced herbicide absorption, reduced translocation, and increased vacuolar sequestration [40,41,42,43], but the metabolism-based weed resistance contributes to significant crop yield losses and quality downgrades, increasing production costs globally.

The high resistance factors for nicosulfuron and rimsulfuron challenge the theory that NTSR typically results in lower resistance factors compared to TSR. However, an NTSR-dominated late watergrass accession with metabolism-based resistance (due to oxidative metabolism via CYP450s) exhibited a 1100-fold resistance factor for the sulfonylurea bensulfuron and a 6.2-fold resistance factor for the triazolopyrimidine penoxsulam [44]. Furthermore, Kaundun [12] reported that the gradual accumulation of NTSR over time in field-selected populations can lead to very high resistance levels, sometimes exceeding those conferred by target-site mechanisms.

The lack of a clear fitness or competitive ability cost in giant foxtail due to enhanced metabolism-based resistance aligns with findings from other studies. In particular, Babineau et al. [45] reported that a loose silky bentgrass population with non-target-site-mediated resistance exhibited no fitness cost in traits such as vegetative biomass and fecundity while also displaying some fitness advantages, including earlier germination and flowering times. Similarly, Keshtkar et al. [46] found no significant differences in fitness traits (potential seed production, vegetative biomass, and tiller number production) in black-grass (*Alopecurus myosuroides* Huds) populations with non-target-site resistance to ACCase inhibitors, whether grown alone or in competition with winter wheat. Vila-Aiub et al. [47], studying seed germination and seedling emergence dynamics in rigid ryegrass populations with different resistance mechanisms (target-site ACCase resistance, P450-based resistance, and ACCase inhibitor susceptibility), reported relatively minor differences between the ACCase-susceptible and CytP450 metabolic-resistant phenotypes, but greater variation when compared to the target-site ACCase inhibitor-resistant phenotype. In addition to non-target-site resistance, absence of fitness and competitive ability penalties has also been reported by Wiederholt and Stoltenberg [48] in giant foxtail populations carrying resistance-endowing mutant alleles to ACCase-inhibiting herbicides. However, Wang et al. [49] found that a green foxtail population carrying the Leu-1781 mutation, which conferred target-site-mediated resistance to ACCase-inhibiting herbicides, exhibited a higher reproductive output compared to the wild-type phenotype. These results indicate that different resistance-conferring alleles and genes can lead to varying fitness effects—negative, neutral, or positive—on field-evolved herbicide-resistant weed populations. This variability means that no generalizations can be made about the fitness costs of non-target-site resistance (NTSR) in grass weed populations, highlighting the need for case-by-case evaluation. Therefore, herbicide rotations with herbicides with different modes of action, mixtures, crop rotation, and cultural control strategies should be designed to delay or mitigate the field selection of resistant populations [50].

## 5. Conclusions

The present study provides strong evidence that five giant foxtail populations from continuous corn monocultures in northeastern Greece have evolved metabolism-based resistance to the ALS-inhibiting herbicides nicosulfuron and rimsulfuron, while remaining susceptible to imidazolinone imazamox, 4-HPPD inhibitor tembotrione, and ACCase inhibitor cycloxydim, suggesting specificity in the resistance mechanism. Sequencing of the *ALS* gene did not detect known resistance-conferring point mutations, indicating that resistance is likely driven by non-target-site mechanisms. This was confirmed by the application of piperonyl butoxide (PBO) prior to nicosulfuron treatment, which successfully reversed resistance, implicating cytochrome P450 monoxygenases in herbicide metabolism and detoxification. Despite the presence of resistance, competition assays revealed no significant vegetative fitness cost in resistant (R) populations. Furthermore, the analysis of the novel *ALS* genotype in *S. faberi* for the first time (submitted to the GenBank database with accession number PV016837) indicated a closer genetic relationship regarding the *ALS* gene between *S. faberi* and *S. viridis* than *S. italica*. Conclusively, these findings highlight the increasing challenge posed by metabolic resistance in giant foxtail populations grown in corn, which can confer cross-resistance to ALS-inhibiting herbicides and limit the effectiveness of current chemical weed control strategies.

## Figures and Tables

**Table 1:** Source of materials for the products used in the screening test and the whole-plant rate-response experiments against the five R and one S giant foxtail populations.

Herbicide ^a^	Trade Name	Form ^b^	Rates	Manufacturer
			g ai ha^−1^	
Nicosulfuron	Samson Extra	OD	5.6	Bayer Crop Science Hellas
			11.2	
			22.5	
			**45** ^c^	
			90	
			180	
			360	
Rimsulfuron	Rush	WDG	1.87	Corteva Agriscience Hellas
			3.75	
			7.5	
			**15** ^c^	
			30	
			60	
			120	
Imazamox	Pulsar^®^	SL	**50** ^c^	BASF Hellas
			100	
Tembotrione	Laudis^®^	OD	**60** ^c^	Bayer Crop Science Hellas
			120	
Cycloxydim	Focus^®^	EC	**200** ^c^	BASF Hellas
			400	

^a^ Rimsulfuron (sulfonylurea, ALS inhibitor) treatments were applied with the surfactant iodecyl alcohol ethoxylate 90% *w*/*v* (Trend^®^ 90 SL, Corteva Agriscience Hellas, Athens, Greece) at 1 ml L^−1^. Imazamox (imidazolinone, ALS inhibitor) was applied with the 37.5% *w*/*w* fatty acid esters + 22.5% *w*/*w* alkoxylated alcohol-phosphate esters (Dash^®^ HC, BASF Hellas, Athens, Greece) at 4 mL L^−1^. ^b^ Abbreviations: OD, oil dispersion; WDG, water dispersible granule. ^c^ The rates in boldface are the label-recommended rates of the herbicides.

**Table 2:** Estimated parameters from the four-parameter log-logistic equations describing the relationship between nicosulfuron or rimsulfuron application rate (g ai ha^−1^) and fresh weight of six giant foxtail populations (one ALS-S and five ALS-R) ^a^.

Parameters	Giant Foxtail Populations					
Nicosulfuron	S (±SE)	R1 (±SE)	R2 (±SE)	R3 (±SE)	R4 (±SE)	R5 (±SE)
b	2.14 ± 0.14 ***	3.12 ± 0.52 ***	2.04	3.54 ± 0.77 ***	2.21 ± 0.37 ***	3.71 ± 0.98 ***
c	−1.41 ± 0.69 ns	69.02 ± 1.75 ***	−49.45	62.57 ± 1.68 ***	55.13 ± 3.68 ***	69.94 ± 2.27 ***
d	100.03 ± 0.67 ***	100.54 ± 1.00 ***	100.71 ± 0.62 ***	99.32 ± 1.28 ***	101.64 ± 1.48 ***	100.08 ± 0.75 ***
e (*GR50*) (g ai ha^−1^)	4.16 ± 0.10 ***	123.64 ± 9.23 ***	703.72	102.65 ± 5.99 ***	115.20 ± 14.22 ***	182.20 ± 10.91 ***
Lower/upper	3.95/4.37	104.66/142.61	-	90.33/114.97	85.97/144.43	159.77/204.63
Rimsulfuron	S (±SE)	R1 (±SE)	R2 (±SE)	R3 (±SE)	R4 (±SE)	R5 (±SE)
b	2.27 ± 0.15 ***	1.75 ± 0.42 ***	1.62 ± 0.31 ***	1.86 ± 0.30 ***	2.34 ± 0.28 ***	3.22 ± 0.38 ***
c	−1.49 ± 0.77 ns	62.25 ± 7.93 ***	53.48 ± 2.39 ***	56.08 ± 2.65 ***	43.98 ± 2.14 ***	50.79 ± 1.96 ***
d	100.19 ± 0.77 ***	100.84 ± 1.26 ***	100.07 ± 1.22 ***	99.78 ± 1.41 ***	98.95 ± 1.21 ***	100.69 ± 1.11 ***
e (*GR50*) (g ai ha^−1^)	1.58 ± 0.03 ***	61.71 ± 17.98 **	17.97 ± 1.56 ***	29.69 ± 3.01 ***	37.10 ± 1.93 ***	43.25 ± 2.22 ***
Lower/upper	1.51/1.65	24.74/98.68	14.76/21.19	23.50/35.88	33.13/41.08	38.70/47.81

**, and ***: Significant at 0.05, 0.01, and 0.001, respectively. ^a^ b: relative slope, c: lower limit, d: upper limit, e: *GR50* value, n.s.: not significant.

**Table 3:** Parameters of the linear equations describing the relationship between corn height or fresh weight and weed density, or height, tiller number, fresh weight, panicle number, and density, of four giant foxtail populations (one ALS-S and three ALS-R).

	Corn	
Populations	Height	Fresh Weight
S	y = −5.2x + 231.5, *R*^2^ = 0.79	y = −30.4x + 738.6, *R*^2^ = 0.83
R1	y = −7.0x + 235.4, *R*^2^ = 0.96	y = −49.9x + 780.6, *R*^2^ = 0.98
R3	y = −5.3x + 232.1, *R*^2^ = 0.78	y = −43.3x + 754.7, *R*^2^ = 0.95
R5	y = −5.8x + 229.7, *R*^2^ = 0.90	y = −49.4x + 773.9, *R*^2^ = 0.98
	Giant Foxtail	
	Height	Fresh Weight
S	y = 4.2x + 62.8, *R*^2^ = 0.96	y = 18.7x − 11.5, *R*^2^ = 0.93
R1	y = 6.7x + 57.5, *R*^2^ = 0.86	y = 12.5x + 1.7, *R*^2^ = 0.93
R3	y = 5.4x + 49.4, *R*^2^ = 0.71	y = 11.9x − 3.3, *R*^2^ = 0.94
R5	y = 4.3x + 66.8, *R*^2^ = 0.79	y = 9.6x + 20.7, *R*^2^ = 0.96
	Tillers	Panicles
S	y = 6.4x − 3.8, *R*^2^ = 0.83	y = 4.9x − 3.3, *R*^2^ = 0.73
R1	y = 5.7x − 2.1, *R*^2^ = 0.95	y = 2.5x − 1.3, *R*^2^ = 0.96
R3	y = 3.7x − 0.7, *R*^2^ = 0.90	y = 2.7x − 1.7, *R*^2^ = 0.91
R5	y = 4.9x + 1.8, *R*^2^ = 0.84	y = 2.1x + 2.3, *R*^2^ = 0.99

## Data Availability

All data generated or analyzed during this study are included in this published article, whereas the novel characterized gene sequence was submitted in the GenBank database and received accession number PV016837.
